# Coordinated Targeting of S6K1/2 and AXL Disrupts Pyrimidine Biosynthesis in PTEN-Deficient Glioblastoma

**DOI:** 10.1158/2767-9764.CRC-23-0631

**Published:** 2024-08-23

**Authors:** Catherine A. Behrmann, Kelli N. Ennis, Pranjal Sarma, Collin Wetzel, Nicholas A. Clark, Kate M. Von Handorf, Subrahmanya Vallabhapurapu, Cristina Andreani, James Reigle, Pier Paolo Scaglioni, Jarek Meller, Maria F. Czyzyk-Krzeska, Ady Kendler, Xiaoyang Qi, Jann N. Sarkaria, Mario Medvedovic, Soma Sengupta, Biplab Dasgupta, David R. Plas

**Affiliations:** 1 Department of Cancer Biology, University of Cincinnati College of Medicine, Cincinnati, Ohio.; 2 Division of Biostatistics and Bioinformatics, Department of Environmental and Public Health Sciences, University of Cincinnati College of Medicine, Cincinnati, Ohio.; 3 Division of Hematology-Oncology, University of Cincinnati College of Medicine, Cincinnati, Ohio.; 4 Department of Veterans Affairs, Cincinnati Veteran Affairs Medical Center, Cincinnati, Ohio.; 5 Department of Pharmacology and Systems Biology, University of Cincinnati College of Medicine, Cincinnati, Ohio.; 6 Department of Pathology and Laboratory Medicine, University of Cincinnati College of Medicine, Cincinnati, Ohio.; 7 UC Brain Tumor Center, University of Cincinnati College of Medicine, Cincinnati, Ohio.; 8 Department of Radiation Oncology, Mayo Clinic, Rochester, Minnesota.; 9 Departments of Neurology and Neurosurgery, Lineberger Comprehensive Cancer Center, University of North Carolina, Chapel Hill, North Carolina.; 10 Division of Oncology, Cincinnati Children’s Hospital Medical Center, Cincinnati, Ohio.

## Abstract

Intrinsic resistance to targeted therapeutics in PTEN-deficient glioblastoma (GBM) is mediated by redundant signaling networks that sustain critical metabolic functions. Here, we demonstrate that coordinated inhibition of the ribosomal protein S6 kinase 1 (S6K1) and the receptor tyrosine kinase AXL using LY-2584702 and BMS-777607 can overcome network redundancy to reduce GBM tumor growth. This combination of S6K1 and AXL inhibition suppressed glucose flux to pyrimidine biosynthesis. Genetic inactivation studies to map the signaling network indicated that both S6K1 and S6K2 transmit growth signals in PTEN-deficient GBM. Kinome-wide ATP binding analysis in inhibitor-treated cells revealed that LY-2584702 directly inhibited S6K1, and substrate phosphorylation studies showed that BMS-777607 inactivation of upstream AXL collaborated to reduce S6K2-mediated signal transduction. Thus, combination targeting of S6K1 and AXL provides a kinase-directed therapeutic approach that circumvents signal transduction redundancy to interrupt metabolic function and reduce growth of PTEN-deficient GBM.

**Significance:**

Therapy for glioblastoma would be advanced by incorporating molecularly targeted kinase-directed agents, similar to standard of care strategies in other tumor types. Here, we identify a kinase targeting approach to inhibit the metabolism and growth of glioblastoma.

## Introduction

Glioblastoma (GBM) frequently arises from oncogenic driver events affecting PTEN, EGFR, and TP53 (1, 2). Proteogenomic and cell-based analyses of GBM showed PTEN inactivation in 51% of primary glioblastomas of all transcriptional subtypes, conferring increased metabolic flux and apoptosis resistance through activation of the phosphatidylinositol-3′-kinase (PI3K)-Akt pathway (3–6). Limited success exists in treating PTEN-deficient GBMs through inhibition of PI3K and downstream mTOR complex 1 (mTORC1), at least partly because of issues of drug bioavailability and systemic toxicities (7–14).

Downstream of PI3K and mTORC1, the ribosomal protein S6 kinase 1 (S6K1), and its paralog S6K2 control protein synthesis, ribosome biogenesis, and metabolic functions (15–19). S6K1 sustains elevated glycolytic flux, fatty acid synthesis, and pyrimidine biosynthesis (17–21). The therapeutic potential of inactivating S6K1 in neoplasia has been established in studies employing genetic targeting strategies. S6K1 genetic inactivation reduced tumor progression in PTEN-deficient leukemia and PTEN-deficient pheochromocytoma, as well as in a model of acute myeloid leukemia (17, 22, 23). A key function of S6K1 is the direct phosphorylation and activation of Carbamoyl-Phosphate Synthetase 2, Aspartate Transcarbamylase, and Dihydroorotase (CAD), which mediates the first step of *de novo* pyrimidine biosynthesis (18, 19). In studies of the metabolic dependencies of PTEN-deficient GBMs, direct metabolic inhibition of the pyrimidine biosynthesis enzyme dihydroorotate dehydrogenase (DHODH) has been shown to induce cytotoxic responses alone or in combination with PI3K inhibition (24, 25). Though not as thoroughly investigated, studies of S6K2 demonstrated a role in regulating apoptosis sensitivity in response to TNFα and TRAIL in breast cancer (26). Together, these data demonstrate the potential therapeutic benefit of targeting S6K1 and S6K2 for counteracting PTEN-deficient tumor growth.

Our previous investigation using a multiple kinase inhibitor, AD80, which inhibits S6K1, revealed the therapeutic potential for targeting S6K1 in PTEN-deficient glioblastoma (27, 28). Kinome-wide analysis of AD80 revealed that targeting S6K1 together with the receptor tyrosine kinase AXL induced PTEN-selective cytotoxic effects in GBM. Consistent with this observation, inhibition of AXL has been shown to enhance the efficacy of PI3K inhibitors in head and neck, as well as esophageal cancers (29). Activation of AXL tyrosine kinase activity is associated with PI3K activation (30). Overexpression of AXL, or its ligand GAS6, is frequently associated with the mesenchymal subtype and poor prognosis in GBM, and both AXL and GAS6 are listed as cancer driver genes in a recent Pan-Cancer CPTAC study (31–34). Previously, we found that combination of the AXL inhibitor BMS-777607 with the S6K1 inhibitor LY-2584702 recapitulated the activity of the multikinase inhibitor AD80 to selectively induce cytotoxic responses in PTEN-deficient GBM cells *in vitro* (28).

Here, we investigated the *in vivo* function and mechanistic underpinnings of combination treatment of PTEN-deficient GBM using the S6K1 inhibitor LY-2584702 and the AXL inhibitor BMS-777607. Results revealed brain-penetrant GBM suppression through combination targeting of S6K1 and AXL. Pharmacogenetic studies showed that LY-2584702 functioned as an effective S6K1 inhibitor while partially inhibiting S6K2 signaling. We found that genetic inactivation of S6K2 increased upstream AXL in GBM cells and gliomaspheres, establishing S6K2 negative feedback control of AXL. Thus, targeting of S6K1/S6K2 requires AXL inhibition in a kinase-directed therapeutic approach that exploits the vulnerability of PTEN-deficient GBM by disruption of pyrimidine biosynthesis.

## Materials and Methods

### Subcutaneous xenograft studies

U87MG GBM cells were cultured as a monolayer in DMEM supplemented with 10% fetal bovine serum, L-glutamine, and Penicillin/Streptomycin. Female nude mice 6 to 8 weeks of age received 1 × 10^6^ cells mixed with Matrigel per manufacturer instructions and were injected subcutaneously in the flank. Treatment began 1 month after implantation when the mean tumor volume was 190 mm^2^ [*V* = *L*(*W*^2^)/2]. Mice were orally gavaged twice daily (BID) with either DMSO, LY-2584702, BMS-777607, or a combination of LY-2584702 and BMS-777607 (12.5 mg/kg each) in vehicle: 4% DMSO and 30% PEG300 in water. Treatment schedule was BID for five consecutive days followed by 2 days with no treatment and continued until experimental endpoints were reached. Statistical analyses were performed using a paired Student *t* test.

### Pharmacokinetics and pharmacodynamics study

Blood–brain barrier penetrance was measured in NSG mice. They were administered 30 mg/kg of LY-2584702, 30 mg/kg BMS-777607, the combination, or DMSO in vehicle (4% DMSO and 30% PEG300 in water) for 3 days BID via oral gavage. One hour after the final dose, mice were euthanized. Time of inhibitor to target was measured using C57BL/6J mice given 30 mg/kg of both LY-2584702 and BMS-777607 or DMSO in vehicle for 3 days BID via oral gavage. Time shown indicates time after final dose at which the mice were euthanized. In both cases, brains were extracted and dissociated in RIPA (supplemented with protease and phosphatase inhibitors) for immunoblotting.

### Immunohistochemical staining of brain tissue

Brains of animals were removed upon euthanization at experimental end points and preserved in 10% Buffered Formalin Acetate for 72 hours. Then, these were transferred to 70% Ethanol and delivered to the UC Histopathology Core Laboratory for processing, embedding, sectioning, and staining.

### Orthotopic xenograft studies

U87MG-GFP-Luc cells were cultured as a monolayer in DMEM supplemented with 10% FBS, L-Q, and Pen/Strep. Female nude mice 6 to 8 weeks of age were intracranially injected with 50,000 U87MG-GFP-Luc GBM cells in saline. Tumors were given 20 days to develop [monitored by bioluminescent imaging (BLI)], and then, mice were divided into two groups: vehicle and drug combination (LY-2584702 + BMS-777607), composed of five mice in each. Mice were orally gavaged twice daily with either DMSO or drug combination (12.5 mg/kg each) in vehicle: 4% DMSO and 30% PEG300 in water. Treatment was carried out for five consecutive days followed by 2 days with no treatment for a total period of 40 days or until experimental endpoints were reached. Luciferase levels were calculated from images taken by a Bruker multispectral FX PRO. Pretreatment BLI shown is from day 14 postimplantation (treatment day −6). Treatment day 32 BLI is 51 days postimplantation. Tumor luciferase signals were compared between the groups. Finally, statistical analyses were performed using a paired Student *t* test.

### Patient-derived xenograft studies

Mayo59 spheres were cultured in Neurobasal-A media supplemented with EGF, FGF, B-27, nonessential amino acids, L-glutamine, Pen-Strep, and Sodium Pyruvate on low adhesion plates. Female NCG mice 6 to 8 weeks of age received 100,000 cells injected into the right cortex. Starting 5 days postinjection, mice were orally gavaged twice daily with either DMSO, LY-2584702, BMS-777607, or a combination of LY-2584702 and BMS-777607 (30 mg/kg each). The vehicle was 4% DMSO and 30% PEG300 in water. Treatment schedule was BID five consecutive days followed by 2 days with no treatment and continued for 12 days. Once experimental endpoints were reached, animals were euthanized. Statistical analyses were performed using a paired Student *t* test and Cox proportional hazard analysis.

### Cell culture and reagents

LN229 and U87MG cells were obtained from the ATCC. U87MG-GFP-Luc cell line was generously gifted from Dr. Atsuo Sasaki’s Lab at the University of Cincinnati. This cell line was cultured in DMEM high glucose supplemented with 10% FBS, Pen/Strep, and L-glutamine. Mayo59 were acquired from the Mayo Clinic GBM PDX database. JHH136 were a generous gift from Dr. Gregory Riggins and Dr. Soma Sengupta. Patient-derived spheres were maintained in low attachment plates. Mayo59 were grown in DMEM-F12 media supplemented with B-27, Pen/Strep, EGF, FGF, and heparin. JHH136 were cultured in NeuroCult NS-A complete media according to manufacturer’s recommendations. Testing for the presence of Mycoplasma and culture identity by STR analysis was performed on a biannual basis. Inhibitors were purchased from Selleckchem.

### AXL ligand stimulation

Cells were stimulated with 400 ng/mL Gas6 and/or 300 nmol/L Phosphatidylserine (PtdSer). Cells were grown in DMEM supplemented with 10% FBS overnight, then serum starved for 6 hours, and then stimulated with PtdSer and/or Gas6 for 45 minutes. Cells were lysed in RIPA buffer supplemented with protease and phosphatase inhibitors for immunoblot studies.

### Western blot

Cells were washed with PBS and then lysed with RIPA buffer (150 mmol/L NaCl, 1% NP-40, 0.5% Sodium Deoxycholate (DOC), 0.1% SDS, 50 mmol/L Tris pH 8.0, and 1 mmol/L EDTA pH 8.0) supplemented with protease and phosphatase inhibitors. Lysates were quantified by BCA. For each sample, ≥20 μg of protein was loaded per lane. Blots were interrogated using the antibodies indicated in Key Resources (Supplementary Table S1) and developed by ECL Prime.

### Generation of CRISPR knockout cells

For virus production, 293T cells were transfected with pLenti Cas9 Blasticidin, pCMV∆R8.2 ∆Vpr (Packaging), and VSV-G envelope to generate Cas9 viral particles. Cells were transduced by introducing the viral supernatant to the target GBM cells at 2,000 × *g* for 60 minutes in the presence of 10 μg/mL polybrene. Cells were later selected with Blasticidin (10 μg/mL)/Hygromycin (10 μg/mL) for 10 to 12 days. Next, viral particles were made in a similar way with guide RNAs and were transduced in Cas9 GBM cells with polybrene (10 μg/mL). Later, the cells were selected with Puromycin (10 μg/mL) for 10 days. Cas9 and guide RNA sequences are listed in Key Resources (Supplementary Table S1). Knockout efficiency was measured by western blot. Guide RNA sequences are given below from the Brunello Library (35):

sgS6K1 (exon 2): 5′-AAT​GAA​AGC​ATG​GAC​CAT​GG

sgS6K1 (exon 5): 5′-AGC​AGA​ACG​GAA​TAT​TCT​GG

sgS6K2 (exon 5): 5′-GGC​CCG​CAC​TCA​TAC​CAC​TG

sgS6K2 (exon 9): 5′-ACT​GCG​CAC​CAG​AAT​CTC​AG

sgNT: 5′- GTA​TTA​CTG​ATA​TTG​GTG​GG

### Adherent cell transfections

LN229 and U87MG-GFP-Luc cells were plated at a density of 200,000 cells per 60 mm plate in DMEM containing 10% FBS, Pen/Strep, and L-glutamine for 24 hours. Cells were transfected with 30 nmol/L siRNAs targeting PTEN, S6K1, or S6K2 in OptiMEM and serum free media using Lipofectamine 3000 reagent for 72 hours. After 72 hours of transfection, cells were plated for respective experiments.

### Gliomasphere electroporation

Mayo59 spheres were treated with Accutase, and then, 500,000 cells in 1.5 μmol/L siRNA were loaded into cuvettes. Next, they were electroporated via the NEPA21 instrument using a Poring Pulse of 150 V, L:5 ms, I:50 ms, 2×, 10%, + and a Transfer Pulse of 20 V, L:50 ms, I:50 ms, 5×, 40%, +/−. Cells were immediately placed into warm growth media and rested for 24 hours. Then, spheres were standardized at 88,000 cells per well and rested for 48 hours. Then, spheres were treated for 3 hours with 10 μmol/L LY-2584702 and/ or 10 μmol/L BMS-777607 in 0.2% DMSO before lysing in RIPA.

### Extreme limiting dilution assay analysis

Gliomaspheres were treated for 72 hours with 10 μmol/L LY-2584702 and/or 10 μmol/L BMS-777607 in 0.2% DMSO. Then, spheres were washed, treated with Accutase, and Trypan Blue negative (live) single cells were plated at 1, 5, 10, or 20 cells per well in 96-well plates in growth media as described (36). After 2 weeks, spheres were stained with 0.5 μmol/L Calcein green and 125 nmol/L Cytotox red, and whole wells were imaged to assess sphere-forming frequency.

### RNA extraction and RNA sequencing

RNA was extracted using TRIzol according to the manufacturer’s instructions. RNA samples were treated with 2 U/μL of rDNase I enzyme in 10× DNase I Buffer and incubated at 37°C for 30 minutes. The reaction was inactivated by adding DNase Inactivation Reagent and then submitted to the Genomics, Epigenomics, and Sequencing Core, University of Cincinnati.

### Analysis for neurosphere image quantification

The FIJI package of ImageJ was utilized for analysis of phase contrast images. Particles larger than 50 μm^2^ were analyzed.

### Metabolic study with LC-MS

LN229 cells were transfected with siPTEN, as described earlier, in DMEM with 0.1% serum for 24 to 48 hours. LN229 or U87MG cells were seeded at a density of 0.2 × 10^6^ per well of a six-well plate in quadruplicate, allowed to grow for 24 to 48 hours in DMEM with 10% serum. LN229 were then incubated either with 0.2% DMSO vehicle control or 10 μmol/L LY-2584702 with 10 μmol/L BMS-777607 for 2 hours in DMEM with 0.1% FBS. Next, LN229 or U87MG cells were incubated with fresh DMEM with 0.1% FBS containing 2 mmol/L glucose and either vehicle control or drug combination for 3 hours. U87MG media was changed to DMEM 0.1% FBS media containing 2 mmol/L U^13^C-glucose and either vehicle control or combination inhibitors and incubated for the indicated timepoints. Media was aspirated, and plates were rinsed twice with ice cold PBS. Samples were extracted using 100 μL of 50:30:20 (MeOH:ACN:H_2_O) and then rocked for 5 minutes in 4°C cold room. Extracts were stored in −80°C for 24 hours, then spun at 13,000 RPM for 5 minutes, and transferred to fresh tubes twice. 20 μL of extract was injected on BEH-amide column (Supplier: Waters) and delivered to the LC-MS Core. Protein was collected from each well for normalization (for absolute abundance). (Mobile Phase A) 10 mmol/L ammonium bicarbonate 0.1% ammonium hydroxide; (Mobile Phase B) Acetonitrile. Extracted ion chromatograms (37) including isotopically labeled peaks were obtained using MAVEN (http://maven.princeton.edu/index.php; refs.^38^, ^39^).

### GSEA methods

We performed Gene Set Enrichment Analysis (GSEA; ref. 40) using the siAXL genome-wide RNAseq signature from Goyette and colleagues (41). 27,108 genes were used as a reference and the signatures from S6K knockdown as queries. We split each S6K knockdown signature into separate lists of up- and down-regulated genes, with lengths ranging from 15 to 183 genes, and queried these lists against the siAXL signature using GSEA. For each score, raw *P* values and *P* values adjusted for FDR are reported.

### Statistical analysis

All data were presented as data points and mean values ± SE. Microsoft Excel and R Project were used to perform data analysis, statistical comparisons by *t* tests, and data plots.

### KiNativ analysis

shPTEN LN229 cells were cultured for 3 hours in DMSO, 10 μmol/L LY-2584702, 10 μmol/L BMS-777607, or combination in DMEM supplemented with 0.1% FBS, glutamine, and Pen/Strep. These were pelleted, flash frozen, and then shipped to ActivX for ATP binding site inhibition analysis. Values shown are relative to DMSO vehicle controls. Kinome tree only reports values of uniquely identifiable kinase domains, created by CORAL (42).

### Key resources table

Descriptions of Key Resources used in the study are available in Supplementary Table S1.

### Data availability

RNAseq data generated in this study are publicly available in Gene Expression Omnibus at GSE196591. Metabolomics and KiNativ ATP binding data are available in Supplementary Tables.

## Results

### Targeted *in vivo* inhibition of S6K1 and AXL in PTEN-deficient GBM tumors

**Figure 1:**
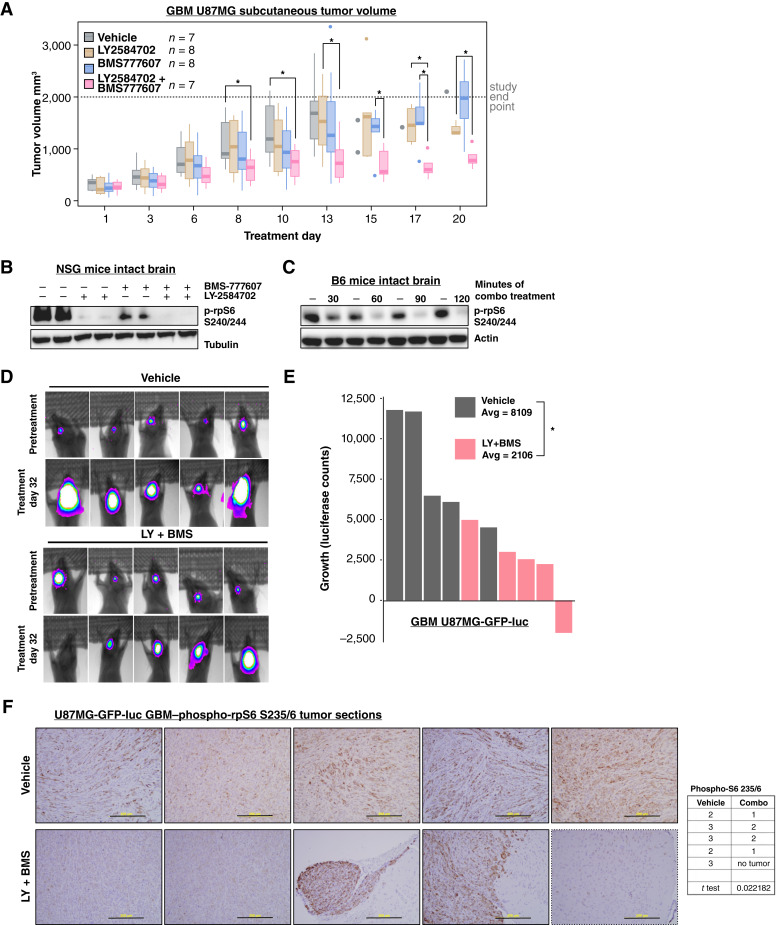
Combination S6K1 and AXL targeting reduces PTEN-deficient glioblastoma tumors *in vivo*. **A,** Combination S6K1 and AXL inhibitors, LY-2584702 and BMS-777607, respectively, reduced subcutaneous tumor volumes. Nude mice were implanted with 1 million U87MG cells and then treated twice daily (BID) with inhibitors by oral gavage on a 5 + 2 schedule. Animals were euthanized once tumors measured 2,000 mm^3^. **B,** Brain lysates from NSG mice treated with 30 mg/kg of LY-2584702, 30 mg/kg of BMS-777607, a combination of LY + BMS, or DMSO for 3 days BID. Reduced rpS6 phosphorylation was detected in intact brains collected an hour after final dose. **C,** In a time course of combination treatment, rpS6 phosphorylation was reduced in B6 mouse intact brain lysates as early as 30 minutes after final treatment. LY-2584702 and BMS-777607 reduced rpS6 phosphorylation in the brain, even in the absence of tumor. **D,** The S6K1 inhibitor, LY-2584702, in combination with the AXL inhibitor, BMS-777607, reduced tumor growth in orthotopically implanted U87MG-GFP-Luciferase cells. Growth of intracranial tumors was determined from bioluminescence imaging before treatment began and again after a month of BID 5 + 2 oral gavage inhibitors (12.5 mg/kg) or vehicle control (*n* = 5). **E,** Tumor growth of **D** shown as the change in luciferase counts from pretreatment to 32 days after treatment initiation. *P* value = 0.015 (*t* test). **F,** Histological staining of phospho-rpS6 (S235/6) of tumor sections from whole excised brains of mice treated with DMSO or LY + BMS combination. The staining intensity of these samples were graded 1–3 in a blinded fashion. Scale bar, 200 μm.

Based on previous findings that combination targeting of S6K1 and AXL effectively reduced viability of PTEN-deficient GBM, we initiated preclinical therapeutic studies by testing inhibitor effects on tumor growth in nude mice that were injected subcutaneously with PTEN-deficient GBM U87MG (27). Mice with palpable tumors were randomized to groups for oral administration of vehicle control, 12.5 mg/kg LY-2584702, 12.5 mg/kg BMS-777607, or a combination of both inhibitors. Tumor volume was significantly reduced in combination-treated mice compared with the vehicle control group at days 8 to 10 of therapy (Fig. 1A; Supplementary Fig. S1A). Although study endpoints were reached within the vehicle control cohort at earlier timepoints, differences in tumor growth between combination and single agent controls became statistically significant at later timepoints (Fig. 1A). Importantly, until tumor endpoints were reached, mice treated with single agents or combination LY-2584702 and BMS-777607 maintained body weight, suggesting that the compounds were well tolerated at effective doses in animals (Supplementary Fig. S1B). Recognizing that U87MG cells are not necessarily a high-fidelity model of GBM because of long passage time and issues related to identification of the tumor of origin, these results are suggestive of the therapeutic potential of combination inhibition of S6K1 and AXL (43).

To determine inhibitor brain penetrance in preparation for orthotopic tumor studies, whole brain lysates were prepared from nontumor-bearing mice treated with single agent 30 mg/kg LY-2584702, single agent 30 mg/kg BMS-777607, or a combination of the inhibitors. Lysates were probed for decreased phosphorylation of the canonical S6K1/S6K2 substrate, ribosomal protein S6 (rpS6). Single agent LY-2584702 strongly suppressed rpS6 phosphorylation, consistent with a recently published predictive analysis of brain penetrance (44), and single agent BMS-777607 also reduced phospho-rpS6, in line with previously described suppression of AXL phosphorylation in brain tumor xenografts (Fig. 1B; ref. 45). Combination of LY-2584702 with BMS-777607 further reduced rpS6 phosphorylation compared with single agents (Fig. 1B). In a time-course study of combination inhibitor treatment, suppression of phospho-rpS6 was sustained for at least 2 hours after drug administration (Fig. 1C). The results indicate effective suppression of signal transduction mediated by the S6 kinases and AXL in mice with an intact blood–brain barrier.

Having established the brain penetrance and efficacy of combined S6K1 and AXL inhibitors in intact brain, the effects of pharmacologic targeting were tested in mice with intracranial implants of the PTEN-deficient GBM U87MG, which was modified to express GFP-Luciferase (U87MG GFP-Luc; ref. 46). Mice bearing established tumors were randomized and treated with vehicle control or combined S6K1 inhibitor LY-2584702 and AXL inhibitor BMS-777607. Combination therapy reduced U87MG tumor growth detected by luciferase imaging (Fig. 1D and E). Further, consistent with the bioluminescence results, intensity scoring of GBM samples stained to detect phospho-rpS6 was reduced, indicating a significant inhibition of S6K signaling (Fig. 1F). Together, these results show that LY-2584702 and BMS-777607 are well tolerated when given orally on a dosing schedule that effectively inhibits rpS6 and reduces GBM tumor size.

### S6K1 and AXL combined inhibition extends survival in patient-derived tumor models

**Figure 2:**
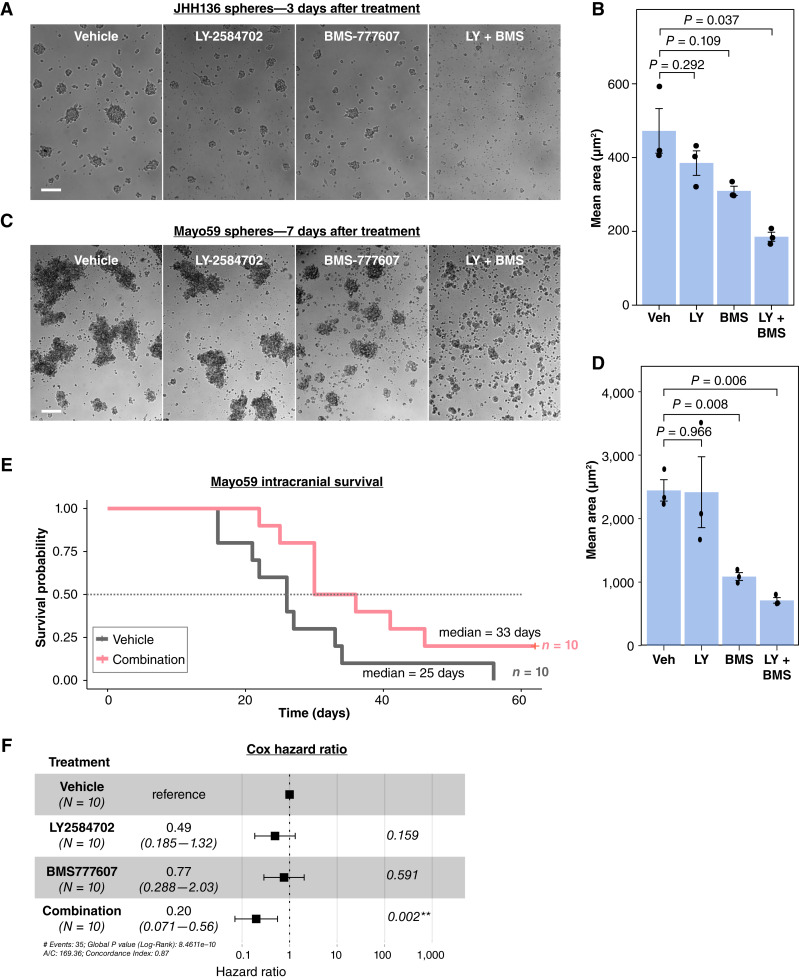
PTEN-deficient patient-derived sphere growth is inhibited by combination S6K1 and AXL inhibition. **A,** JHH136 patient-derived GBM were treated for 72 hours with 10 μmol/L LY-2584702 and/ or 10 μmol/L BMS-777607. Phase contrast images show reduced sphere size with inhibitor combination compared with single inhibitor treatment and vehicle control (*n* = 3). Scale bar, 100 μm. **B,** Quantification of spheres in three independent cultures as in **A** are shown. *P* values from *t* tests indicate combination efficacy compared with single agent controls. **C,** Mayo59 treated with 10 μmol/L LY-2584702 and/or 10 μmol/L BMS-777607 for 7 days. Combination treatment reduced sphere size and induced an altered morphology (*n* = 3). Scale bar, 200 μm. **D,** Quantification of images in **C** shows significant reduction in sphere size in combination-treated spheres. **E,** Symptom-free survival of mice bearing intracranial tumors of Mayo59 PDX treated with vehicle or a combination of LY-2584702 and BMS-777607 together. Survival curve shows that animals gained a sustained delay in disease from combination treatment (*n* = 10 per group). **F,** Cox proportional hazard ratio analysis for mice in **E**. Log-rank score for combination treatment is below 0.002.

To develop combination targeting of S6K1 and AXL in a PTEN-deficient patient-derived low-passage glioblastoma model, we investigated the effects of treatment with the S6K1 inhibitor LY-2584702 in combination with the AXL inhibitor BMS-777607 in gliomaspheres, which can better preserve GBM tumor-propagating cellular states (47, 48). We tested LY-2584702 in combination with BMS-777607 using a gliomasphere line GBM59 (here designated Mayo59), which was selected because of its deep deletions in the PTEN locus, as well as gliomasphere line JHH136, which is an established PTEN-deficient gliomasphere line (49, 50). Treatment with the S6K1 inhibitor LY-2584702 resulted in a trend toward reduced gliomasphere mean area measured in images from triplicate cultures of JHH136 (Fig. 2A and B) and Mayo59 (Fig. 2C and D). Single agent AXL inhibitor BMS-777607 was sufficient to reduce mean area, and the combination of LY-2584702 with BMS-777607 significantly reduced the mean gliomasphere area measured on day 3 and 7 of treatment (Fig. 2A–D). To investigate potential effects in reducing the frequency of stem-like tumor-propagating cells, we conducted extreme limiting dilution analysis in Mayo 59 and JHH-136 gliomaspheres that were treated with vehicle, single agents, and combination kinase inhibitors for 3 days (51). Extreme limiting dilution analysis did not reveal statistically significant differences in response to combination inhibitor treatment, indicating that a preferential selectivity of LY-2584702 and BMS-777607 in depleting the percentage of stem-like cells was not found compared with bulk sphere cells in other cellular states (Supplementary Fig. S2A and S2B).

To determine if combination kinase targeting affected GBM survival, PTEN-deficient Mayo59 gliomaspheres were selected for intracranial tumor modeling, considering the magnitude of the reduction in gliomasphere mean area (Fig. 2C and D). After intracranial placement of Mayo59 cells, mice were randomized into groups to be treated with single or combination agents. LY-2584702 at 30 mg/kg and/or BMS-777607 at 30 mg/kg was delivered to recipient mice for two courses of five consecutive days, separated by an interval of 2 days, via oral gavage, as indicated (45, 52). Next, median survival of mice treated with combination therapy was 33 days compared with 25 for vehicle control, an increase of 32% (Fig. 2E). No survival benefit was found in mice treated with single agents (Supplementary Fig. S2C). Analysis of survival by Cox Proportional Hazards to model treatment effects revealed a significant survival advantage in the combination treatment group compared with vehicle and single drugs (Fig. 2F; Supplementary Fig. S2D). Also, injection day and body weight were analyzed using Cox Proportional Hazards, with day of injection showing significant differences that did not impact the effects of treatment (Supplementary Fig. S2D). Altogether, *in vivo* tumor growth studies show that LY-2584702 and BMS-777607 are orally bioavailable, brain-penetrant, and effective in combination to inhibit PTEN-deficient GBM tumor growth.

### Combination therapy reduces pyrimidine biosynthesis in PTEN-deficient GBM

**Figure 3:**
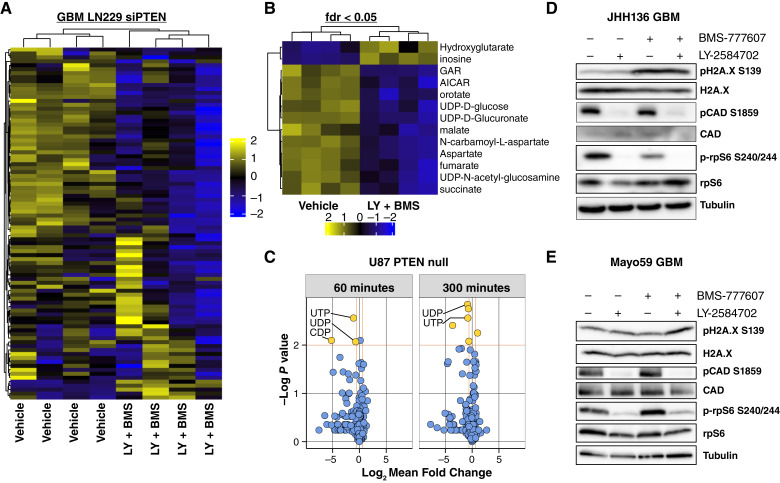
S6K1 and AXL inhibitors counteract pyrimidine biosynthesis in PTEN-deficient GBM. **A,** Steady state metabolite abundance in LN229 GBM transfected with siPTEN and then treated with vehicle control or combination S6K1 (LY-2584702, 10 μmol/L) and AXL (BMS-777607, 10 μmol/L) inhibitors for 5 hours (*n* = 4). **B,** Detail of nucleotide and their precursor metabolites from **A**. **C,** log_2_ fold change of [U]-^13^C glucose labeled metabolites in U87MG GBM pretreated for 3 hours with 10 μmol/L LY-2584702 and 10 μmol/L BMS-777607 vs. vehicle control at 60 and 300 minutes after addition of ^13^C-glucose (*n* = 4). Statistically significant (>1.5 fold) metabolites are highlighted. **D,** JHH136 spheres were treated with inhibitors (10 μmol/L each) for 72 hours for western blot analysis. LY-2584702, S6K1 inhibitor, reduces phosphorylation of rpS6 and CAD leading to sustained impairment of pyrimidine synthesis and cell growth. Treatment with BMS-777607 increases H2A.X phosphorylation at Serine 139, indicating an increase in double-stranded DNA breaks. **E,** Mayo59 spheres were treated as in **D**. S6K1 inhibition reduces phosphorylation of rpS6 and CAD, impairing pyrimidine synthesis. DNA damage is evident in combination S6K1 and AXL inhibition.

PTEN deficiency drives increased metabolic flux supporting bioenergetic and biosynthetic functions of transformed cells (53, 54). Downstream of PTEN, S6K1 is required to mediate elevated glucose flux through the glycolysis pathway (17). Additional S6K1 roles in oncogenic metabolism include the induction of pyrimidine biosynthesis through the direct phosphorylation of CAD (18, 19) and promotion of lipid synthesis through increased processing of SREBP-1 (55). To explore the interaction between PTEN loss and inhibitor treatment on the metabolome, metabolite abundance was profiled in PTEN-knockdown LN229 GBM cells treated with combination LY-2584702 and BMS-777607 inhibitors. A decreased abundance of the pyrimidine precursor metabolites aspartate and N-carbamoyl aspartate was found upon treatment with combination LY-2584702 and BMS-777607 (Fig. 3A and B; Supplementary Table S2), consistent with a requirement for S6K1 in promoting pyrimidine biosynthesis. To study glucose flux in constitutively PTEN-deficient cells treated with kinase inhibitors, U87MG GBM were treated with LY-2584702 and BMS-777607 for three hours and then pulsed with [U]-^13^C-glucose. Results revealed decreased incorporation of glucose-derived carbon into pyrimidines at 60 and 300 minutes in inhibitor-treated cells (Fig. 3C; Supplementary Table S3). This indicates that combination drug treatment induced an acute and sustained deficiency in pyrimidine biosynthesis. In addition to decreased ^13^C-labeling of the uridine and cytidine pyrimidines, a decrease in ^13^C-labeling of UDP-modified carbohydrates was detected (Supplementary Fig. S3), indicating that reduced pyrimidine biosynthesis impacted downstream metabolite pools. To determine the possible impact of metabolic alterations in response to combined kinase inhibition in low-passage gliomasphere models, we tested the S6K1-dependent phosphorylation of the pyrimidine biosynthesis enzyme CAD. In PTEN-deficient gliomaspheres, decreased CAD phosphorylation at Ser1859 was observed upon treatment with LY-2584702 single agent or in combination with BMS-777607, consistent with the role of S6K1 in the control of CAD-dependent pyrimidine biosynthesis (Fig. 3D and E; refs. 18, 19). Also, we observed induction of Ser139 phosphorylation of histone H2A.X (also described as γH2A.X), a marker of DNA damage. BMS-777607 treatment corresponded with induction of H2A.X S139 phosphorylation in JHH136 gliomaspheres and Mayo59 increased H2A.X S139 phosphorylation upon treatment with both inhibitors (Fig. 3D and E). Altogether, these data indicate that combination LY-2584702 and BMS-777607 reduces pyrimidine biosynthesis while potentiating DNA damage signaling in GBM.

### S6K1 and S6K2 mediate signaling activated by PTEN loss

**Figure 4:**
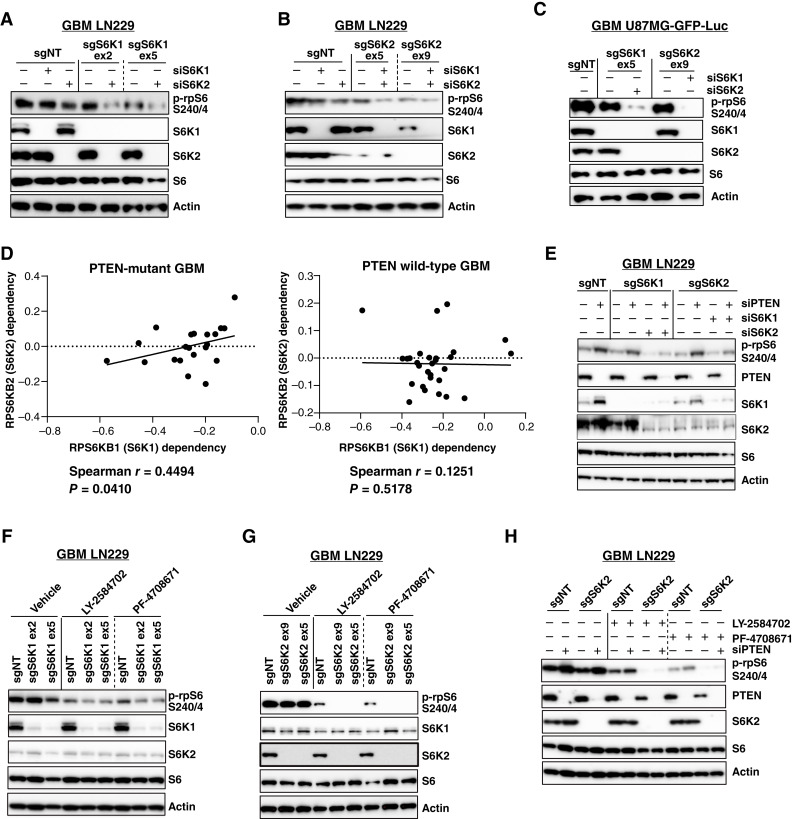
Targeting requirements in the S6K1/S6K2 network. **A** and **B,** S6 phosphorylation was reduced only upon genetic silencing of both S6K1 and S6K2 in LN229 GBM cells. **C,** Combined inactivation of S6K1 and S6K2 via sgRNA and siRNA was required to silence S6K signaling in PTEN-deficient GBM U87MG-GFP-Luc. **D,** DepMap correlation shows dependence on S6K1 and S6K2 when PTEN is mutated. **E,** PTEN inactivation caused an induction of S6K signaling seen as an increase in the phosphorylation of rpS6. This increase can only be abrogated by combination inactivation of both S6K1 (exon 5) and S6K2 (exon 9). **F,** sgNT and sgS6K1 GBM LN229 cells were treated with 10 μmol/L LY-2584702 or 10 μmol/L PF-4708671 for 3 hours. Inhibitor suppression of phospho-rpS6 was modestly improved by knockout of S6K1. **G,** sgNT and sgS6K2 GBM LN229 cells were treated as in **F**. Inhibitor suppression of phospho-rpS6 was significantly enhanced by S6K2 knockout. **H,** sgNT and sgS6K2 (exon 9) LN229 cells transfected with siNT or siPTEN for 72 hours were incubated with S6K1 inhibitors for 3 hours. PTEN inactivation induced phospho-rpS6, which was blocked by the combination of sgS6K2 and an S6K1 inhibitor.

Genetic knockout studies showed that both S6K1 and S6K2 can mediate rpS6 phosphorylation, suggesting that pharmacologic strategies to counteract PTEN loss in GBMs require suppression of both paralogs (15). To directly test this hypothesis, the roles of S6K1 and S6K2 in mediating rpS6 phosphorylation were assessed using genetic inactivation studies in LN229 and U87MG-GFP-Luc GBM. LN229 cells were tested to determine the specific effects of PTEN using genetic inactivation, and U87MG cells were tested as a model of sustained PTEN absence. Single sgRNA inactivation of S6K1 or S6K2 modestly reduced rpS6 phosphorylation at Ser240/244 in both LN229 and U87MG GBMs (Fig. 4A–C). Combined sgRNA inactivation of S6K1 and S6K2 did not yield stable cell populations lacking both kinases, despite multiple attempts. Thus, to assess the effect of combined inactivation of both S6K1 and S6K2, we transfected siRNA duplexes targeting S6K1 or S6K2 in established, stable knockout populations. Although single knockout or knockdown of S6K1 or S6K2 was insufficient to entirely reduce rpS6 phosphorylation, the combination of S6K1 and S6K2 inactivation cooperated to substantially reduce rpS6 phosphorylation, consistent with overlapping and compensatory functions in GBM (Fig. 4A–C). Interestingly, analsyis in DepMap supports that dependency of cells on both S6K1 and S6K2 is correlated solely in PTEN-deficient GBMs (Fig. 4D; ref. 56). To test whether S6K1 or S6K2 mediated increased signaling upon PTEN loss, we compared the effects of S6K1/2 inactivation in cells transfected with nontargeting or PTEN siRNA. The absence of single S6K1 or S6K2 paralogs was insufficient to block induction of phospho-S6 upon PTEN loss, but a combination of S6K1 and S6K2 inactivation blocked increases in rpS6 phosphorylation (Fig. 4E). These results establish that combined inactivation of both S6K1 and S6K2 kinases is required to prevent oncogenic signaling induced by PTEN inactivation.

### S6K2 determines the efficacy of pharmacologic S6K1 and AXL targeting

Compared with S6K1 or S6K2 knockout cells that maintain wild-type levels of rpS6 phosphorylation, the S6K1 inhibitors LY-2584702 and PF-4708671 were partially effective in reducing phospho-rpS6 (Fig. 4F and G). To map the mechanism of S6K1 inhibitor functions, we tested responses to inhibitors using GBM cells genetically deficient in S6K1 or S6K2. Only a slight reduction of phospho-rpS6 in sgS6K1 cells treated with LY-2584702 or PF-4708671 was found, consistent with their activity as effective S6K1 inhibitors (Fig. 4F). Interestingly, in sgS6K2 cells, the reduction of phospho-rpS6 in response to LY-2584702 or PF-4708671 was significantly enhanced (Fig. 4G). Considering that LY-2584702 and PF-4708671 were more effective than sgS6K1 in reducing rpS6 phosphorylation and that genetic inactivation of S6K2 potentiated rpS6 suppression, these results indicate that both agents are highly effective S6K1 inhibitors that demonstrate additional partial activity for interfering with S6K2. In cells with PTEN genetic inactivation, single agent S6K1 inhibitors partially reduced rpS6 phosphorylation, whereas combination of inhibitors with S6K2 genetic inactivation enabled the full suppression of phospho-rpS6 (Fig. 4H). Thus, pharmacogenetic analysis indicates that LY-2584702 and PF-4708671 effectively counteract S6K1 signaling while partially interfering with S6K2 signaling.

### S6K2 determines GBM responses to kinase-targeted therapy

**Figure 5:**
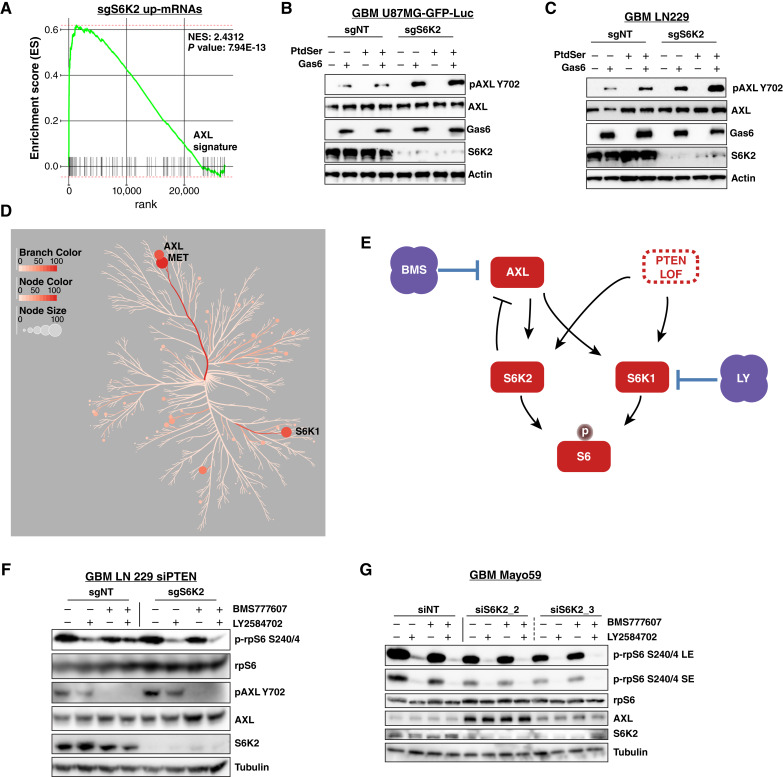
S6K2 and AXL have an effector—regulator relationship. **A,** GSEA identified an AXL response signature in RNA sequence analysis of sgS6K2 LN229 GBM cells. **B** and **C,** sgNT or sgS6K2 (exon 9) GBM U87MG-GFP-Luc (**B**) or LN229 (**C**) cells were stimulated with 300 nmol/L Phosphatidylserine (PtdSer) and/or 400 ng/mL Gas6 for 45 minutes as indicated. AXL autophosphorylation of pY702 was increased in the absence of S6K2. **D,** KiNativ analysis represented as a dendrogram (CORAL kinome tree) in which kinase inhibition in combination-treated shPTEN LN229 cells compared with vehicle is expressed as increased node size, branch intensity, and node intensity. Combination treatment with 10 μmol/L LY-2584702 and 10 μmol/L BMS-777607 for 3 hours specifically targeted S6K1, AXL, and MET. **E,** Model showing S6K2 as both a signaling effector and a feedback regulator of AXL. **F,** siPTEN LN229 sgNT or sgS6K2 cells were treated for 3 hours with 10 μmol/L LY-2584702 and/or 10 μmol/L BMS-777607 as indicated. S6K2 genetic deletion increased AXL phosphorylation. Combination of LY-2584702 with BMS-777607 was required to reduce both rpS6 and AXL phosphorylation. **G,** Mayo59 gliomaspheres were transfected with siNT or independent siS6K2 duplexes prior to treatment with 10 μmol/L LY-2584702 and/or 10 μmol/L BMS-777607 for 3 hours as indicated. Treatment with LY-2584702 reduced rpS6 phosphorylation and S6K2 genetic inactivation resulted in increased AXL, consistent with the requirement for combination with BMS-777607 in reducing GBM growth.

Considering the partial inactivation of S6K2 by LY-2584702, we determined the functional impact of S6K2 inactivation on AXL by GSEA of RNA sequencing data from GBM LN229. GSEA revealed a significant association of S6K inactivation with an siAXL signature, with the most statistically significant associations observed in response to inactivation of S6K2 (Fig. 5A; Supplementary Fig. S4A and S4B). AXL is partially activated in response to the protein ligand GAS6, whereas full AXL activation is achieved by GAS6 that is assembled in complex with phosphatidylserine (PtdSer; refs. 57–59). In U87MG and LN229 GBM cells with stable knockout of S6K2, AXL activation in response to both GAS6 and GAS6:PtdSer was substantially increased compared with control cells (Fig. 5B and C). In contrast to S6K2 inactivation, sgS6K1 induced little effect on AXL in either baseline or ligand-stimulated conditions (Supplementary Fig. S4C). To map the effects of LY-2584702 and BMS-777607 in the GBM kinome, we determined the ATP binding activity of kinases in lysates of GBM LN229 shPTEN cells treated with single agents or combination inhibitors for 3 hours using KiNativ analysis. Results showed decreased ATP loading activity in S6K1 in response to LY-2584702 and suppression of ATP binding activity of AXL and MET in response to BMS-777607 (Fig. 5D; Supplementary Fig. S4D; Supplementary Table S4). Considering the partial activity of LY-2584702 in interfering with S6K2 signaling (Fig. 4G and H) and the priming of AXL signaling by S6K2 genetic loss (Fig. 5B and C), we considered that LY-2584702 and BMS-777607 is an effective combination by circumventing a negative feedback loop between AXL and S6K2 (Fig. 5E). Consistent with this model, only the combination of LY-2584702 and BMS-777607 could silence phospho-rpS6 while also preventing AXL activation in PTEN deleted GBM LN229 cells (Fig. 5F). In Mayo59 gliomaspheres, transient S6K2 inactivation using two independent siRNA duplexes increased AXL protein, and thus combination of LY-2584702 with BMS-777607, is an effective strategy to circumvent feedback activation of AXL (Fig. 5G). Together, the data show that suppression of GBM growth through combination S6K1 and AXL targeting is achieved by preventing the activation of upstream AXL under feedback control by S6K2, which is sufficient to sustain GBM pyrimidine biosynthesis and growth.

## Discussion

Investigating the therapeutic potential for pharmacologic targeting of S6K1 in PTEN-deficient glioblastoma, we previously found that co-targeting AXL with S6K1 induced cytotoxic responses selectively in PTEN-deficient glioblastoma cells (28). Here, results shown demonstrate brain penetrance and therapeutic response to combination S6K1 and AXL inhibitors *in vivo*.

Combination targeting of S6K1 and AXL interrupts signaling to the canonical S6K1/S6K2 substrate rpS6. Detailed analysis of signaling responses to treatment with S6K1 and AXL inhibitors unveiled a key role for S6K2 in mediating kinase signaling to downstream substrates. Additionally, genetic S6K2 inactivation primed compensatory upstream AXL activation, revealing a need for simultaneous targeting of both S6K1 and AXL.

Inhibition of S6K1 using LY-2584702 has been tested in Phase 1 clinical trials for solid tumors. Grade 3 dose-limiting toxicities were observed in patients treated with single agent LY-2584702 and in patients treated with LY-2584702 in combination with the EGFR inhibitor erlotinib (NCT01394003, NCT01115803; refs. 60, 61). Phosphorylation of rpS6 was used as a biomarker for LY-2584702 efficacy in clinical trial skin biopsy samples, and yet, data shown here indicate that LY-2584702 is not necessarily effective as a single agent for suppressing rpS6 phosphorylation because of the partial inhibitory activity of LY-2584702 against S6K2, which must be targeted to reduce rpS6 phosphorylation (Figs. 4A, B, G, H, and 5D; Supplementary Fig. S4D). These results indicate that LY-2584702 and related compounds should be reevaluated for on-target efficacy and for potential combination with tyrosine kinase inhibitors. The tyrosine kinase inhibitor BMS-777607, also known as ASLAN-002, has been tested as a single agent in a Phase 1 clinical trial involving patients with advanced or metastatic solid tumors (NCT01721148; ref. 62). At the recommended Phase 2 dose of 300 mg BID, the compound was found to be well tolerated, with adverse events primarily related to nausea, fatigue, and constipation (62). Interestingly, BMS-777607 also showed potential efficacy in preclinical studies in combination with PD-1-targeting immune checkpoint blockade (63). Pan-cancer whole genome analysis identifies PTEN among the top five genes most frequently affected by cancer driver events (64). Our results warrant further development of S6K1-targeting strategies in combination with AXL inhibition to overcome signaling redundancy mediated by S6K2 in GBM and other cancers frequently affected by PTEN inactivation.

The metabolic signature of S6K1 and AXL combinated inhibition was highlighted by reduced biosynthesis of pyrimidines. Pyrimidine biosynthesis has been previously identified as a metabolic vulnerability in PTEN-deficient glioblastoma (24), and pyrimidine biosynthesis inhibitors have been shown to potentiate the therapeutic response to PI3K inhibitors in glioblastoma (25). Next, pyrimidine biosynthesis has been recently demonstrated to produce required substrates to maintain overall central carbon metabolism and lipid biosynthesis, suggesting the potential therapeutic benefit that may be attained in strategies that reduce cancer cell pyrimidine synthesis (65). Nevertheless, neither pyrimidine biosynthesis inhibitors nor PI3K inhibitors advanced beyond clinical trials for glioblastoma (see NCT00003293, NCT00704080, NCT03696355, NCT05009992, NCT02430363, NCT01240460, clinicaltrials.gov). By circumventing redundancy in signaling between S6K1/2 and AXL, combination LY-2584702 and BMS-777607 treatment presents a novel kinase-directed approach to capitalize on the vulnerability of PTEN-deficient glioblastomas to inhibit pyrimidine biosynthesis.

## Supplementary Material

Figure S1 Tumor growth and body weight

Figure S2 Gliomasphere drug responses

Figure S3 Glucose flux

Figure S4 GSEA, Kinativ

Supplementary Table S1 Key Resources

Supplementary Table S2 Metabolites

Supplementary Table S3 Glucose flux

Supplementary Table S4 Kinativ
