# Supplementary material for: Coordinated Targeting of S6K1/2 and AXL Disrupts Pyrimidine Biosynthesis in PTEN-Deficient Glioblastoma
Source: Cancer Res Commun. 2024 Aug 23;4(8):2215–27. doi: 10.1158/2767-9764.CRC-23-0631 (PMC11342319; doi:10.1158/2767-9764.CRC-23-0631)
Supplement: Figure S1 — Tumor growth and body weight [file crc-23-0631_figure_s1_supps1.pdf]

A

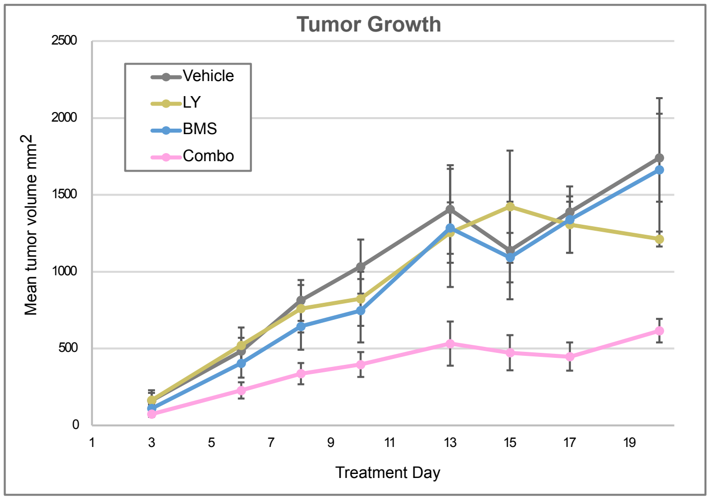

B

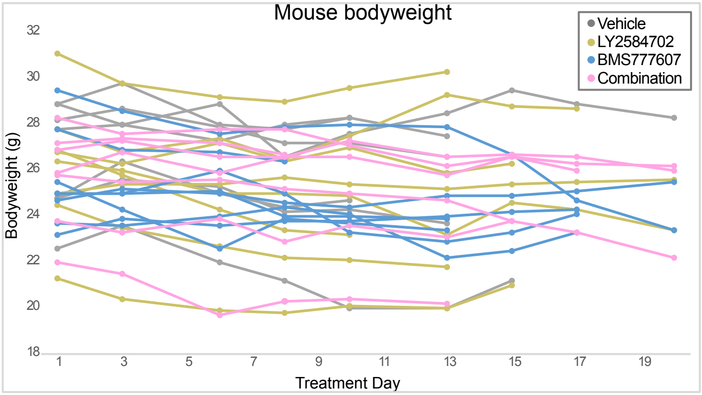

Supplementary Figure 1. *In vivo* inhibitor effects.
(**A**) Tumor growth change from volume at treatment onset. (**B**) Body weight (grams) of mice treated with DMSO, 12.5mg/ kg LY-2584702, 12.5mg/ kg BMS-777607, or a combination of LY and BMS in vehicle (4% DMSO and 30% PEG300 in water). Animals tolerated the twice daily (BID) treatment "5+2 schedule" well.
