# Supplementary material for: Coordinated Targeting of S6K1/2 and AXL Disrupts Pyrimidine Biosynthesis in PTEN-Deficient Glioblastoma
Source: Cancer Res Commun. 2024 Aug 23;4(8):2215–27. doi: 10.1158/2767-9764.CRC-23-0631 (PMC11342319; doi:10.1158/2767-9764.CRC-23-0631)
Supplement: Figure S2 — Gliomasphere drug responses [file crc-23-0631_figure_s2_supps2.pdf]

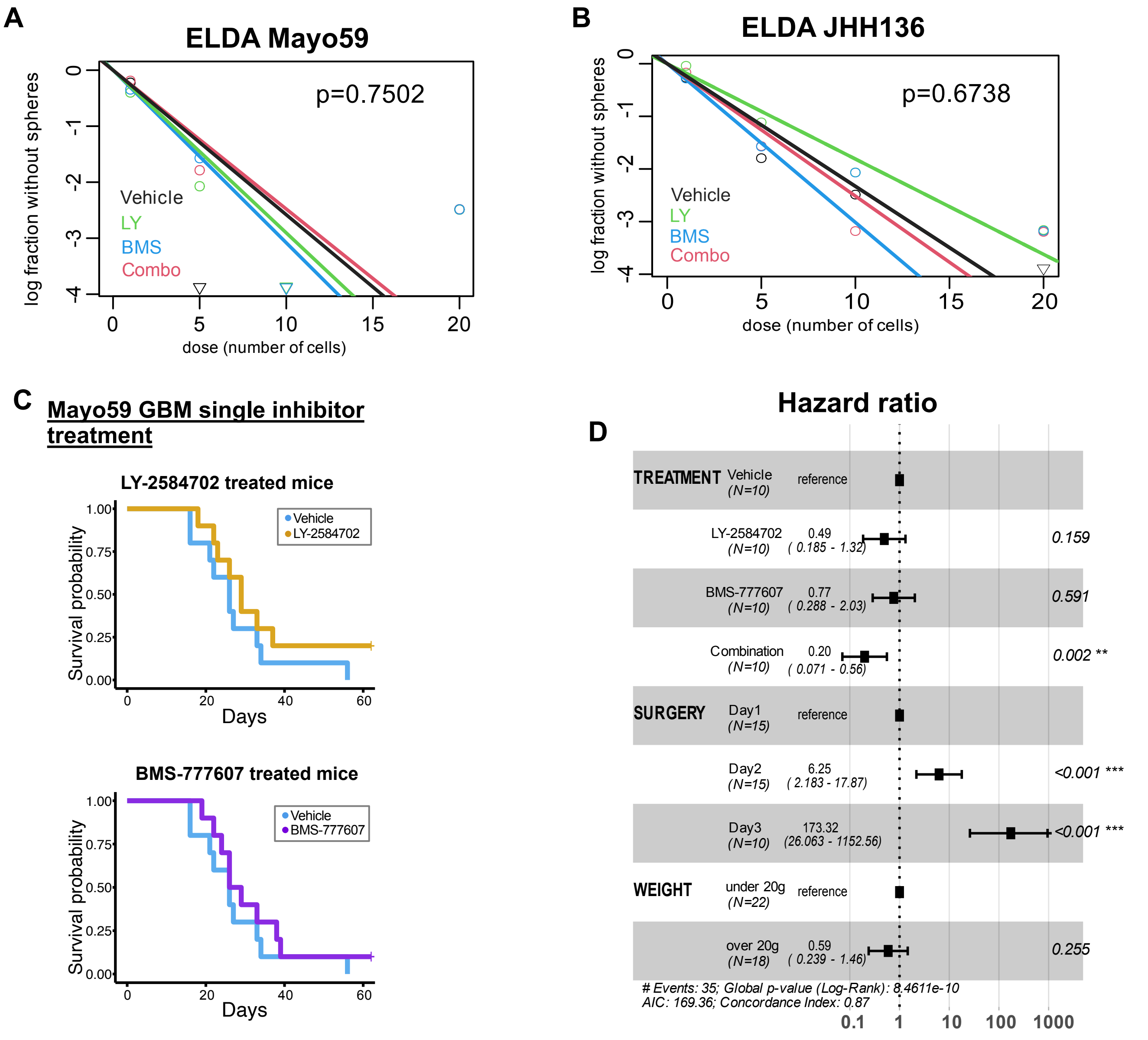

Supplementary Figure 2. Orthotopic patient-derived spheres with S6K1 and AXL inhibition. ((**A and B**) Mayo59 (A) and JHH136 (B) gliomaspheres were treated with 10 µM LY-2584702, 10 µM BMS-777607, or combination kinase inhibitors for 72 hours as indicated. After dissociation to single cell suspensions, gliomasphere-formation activity was assessed by ELDA. Chi-squared statistical analysis did not reveal significant differences. (**C**) Kaplan-Meier curves of animals with intracranial Mayo59 tumors treated with single agent LY-2584702 or BMS-777607. There is no survival advantage to animals with single agent treatment. (**D**) Calculated Cox proportional hazard ratio for animals with intracranial Mayo59 tumors. There is a significant (p=0.002) difference in survival between vehicle and combination treated animals. Surgery day also showed significant survival changes, though this did not alter the treatment effect. Weight of animals did not influence survival in this study.
