# Supplementary material for: Coordinated Targeting of S6K1/2 and AXL Disrupts Pyrimidine Biosynthesis in PTEN-Deficient Glioblastoma
Source: Cancer Res Commun. 2024 Aug 23;4(8):2215–27. doi: 10.1158/2767-9764.CRC-23-0631 (PMC11342319; doi:10.1158/2767-9764.CRC-23-0631)
Supplement: Figure S3 — Glucose flux [file crc-23-0631_figure_s3_supps3.pdf]

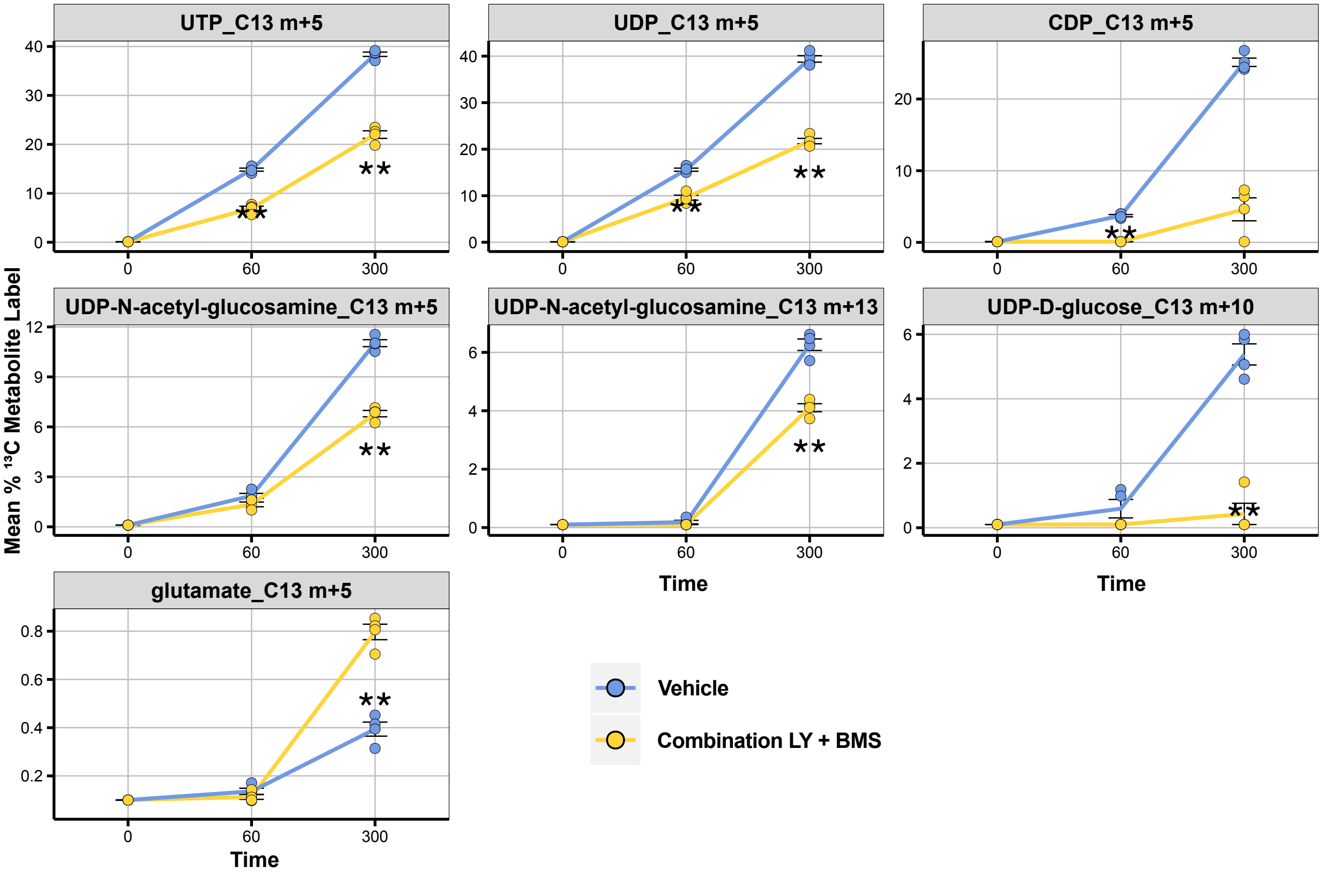

Supplementary Figure 3: **Combination of LY-2584702 and BMS-777607 impairs pyrimidine synthesis.**

Time course of U87MG cells treated with 10μM LY- 2584702 and 10μM BMS-777607. (n=4) Flux at ^{13}C-glucose is shown for the labeled metabolite pools. Statistically significant differences by t-test, corrected for multiple hypothesis testing, are shown. ** $p < 0.01$
