# Supplementary material for: Coordinated Targeting of S6K1/2 and AXL Disrupts Pyrimidine Biosynthesis in PTEN-Deficient Glioblastoma
Source: Cancer Res Commun. 2024 Aug 23;4(8):2215–27. doi: 10.1158/2767-9764.CRC-23-0631 (PMC11342319; doi:10.1158/2767-9764.CRC-23-0631)
Supplement: Figure S4 — GSEA, Kinativ [file crc-23-0631_figure_s4_supps4.pdf]

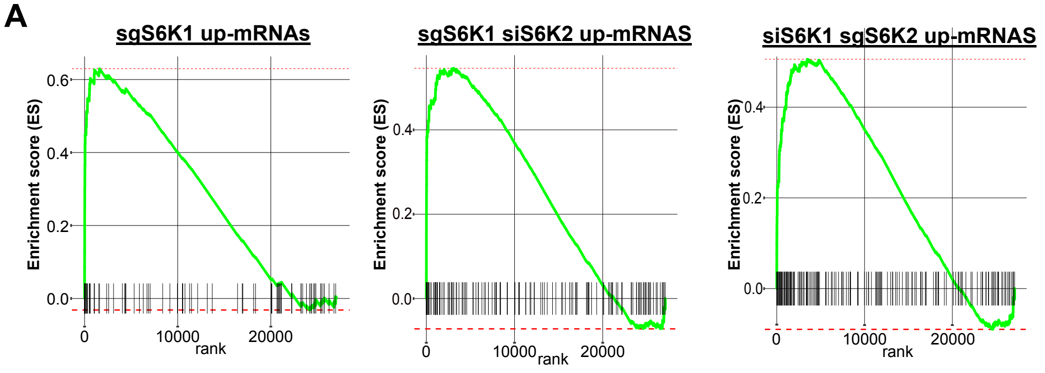

B

Experiment	Regulation	P-value	P-adjusted
S6K2 CRISPR	up	7.93734E-13	9.52481E-12
S6K1 CRISPR	up	5.69922E-11	2.27969E-10
S6K1-CRISPR S6K2-RNAi	up	4.30589E-11	2.27969E-10
S6K1-RNAi S6K2-CRISPR	up	7.46912E-10	2.24074E-09
S6K1 RNAi	down	0.009125956	0.021902294
S6K1-CRISPR S6K2-RNAi	down	0.011271033	0.022542065
S6K2 RNAi	down	0.084070796	0.144121365
S6K1-RNAi S6K2-CRISPR	down	0.107594937	0.161392405
S6K1 RNAi	up	0.192371476	0.209859792
S6K2 RNAi	up	0.175347222	0.209859792
S6K1 CRISPR	down	0.176119403	0.209859792
S6K2 CRISPR	down	0.393063584	0.393063584

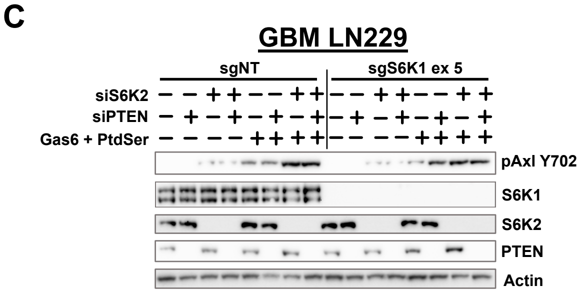

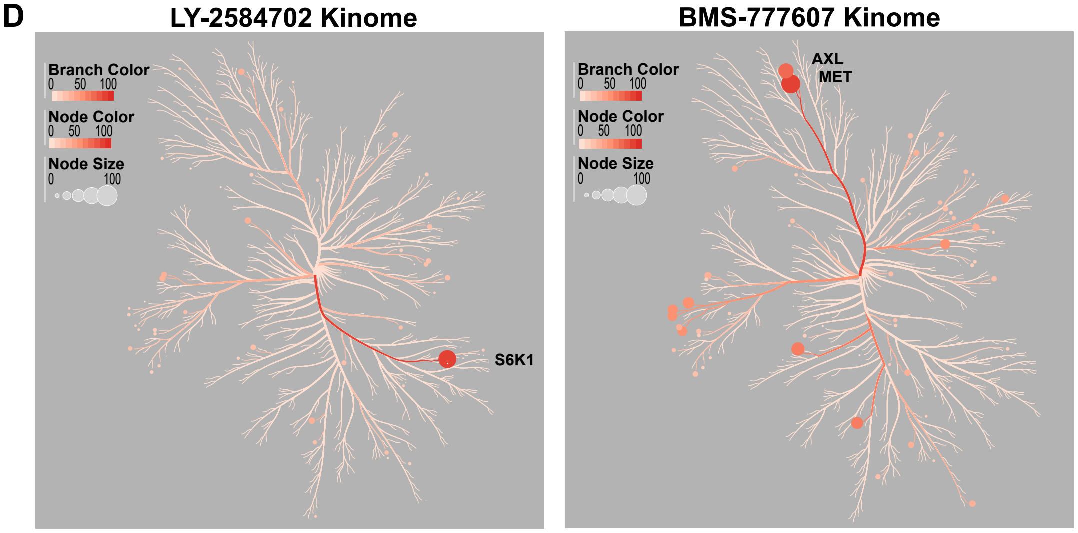

Supplementary Figure 4. S6K2, and not S6K1, interacts with AXL.
(**A**) Gene Set Enrichment Analysis of RNA sequencing data from sgS6K1, sgS6K1+siS6K2, and siS6K1+sgS6K2 LN229 cells. (**B**) Gene set enrichment scores for the AXL signature for all combinations of S6K1 and S6K2 inactivation studied. (**C**) sgNT and sgS6K1 LN229 GBM transfected with siRNA against S6K2 and/ or PTEN then stimulated with a combination of 300nM Phosphatidylserine (PtdSer) and 400ng/mL of Gas6 for 45 minutes. The genetic deletion of S6K1 does not increase AXL phosphorylation above S6K2 deletion levels in the unstimulated or stimulated condition. (**D**) Kinome trees of LY-2584702 or BMS-777607 representing results from a KiNativ assay of LN229 GBM shPTEN cells treated with either inhibitor for 3 hours at 10μM each. Related to Figure 5.
