# Supplementary material for: Coordinated Targeting of S6K1/2 and AXL Disrupts Pyrimidine Biosynthesis in PTEN-Deficient Glioblastoma
Source: Cancer Res Commun. 2024 Aug 23;4(8):2215–27. doi: 10.1158/2767-9764.CRC-23-0631 (PMC11342319; doi:10.1158/2767-9764.CRC-23-0631)
Supplement: Supplementary Table S1 — Key Resources [file crc-23-0631_supplementary_table_s1_suppst1.docx]

Supplementary Table 1. Key Resources

| **REAGENT OR RESOURCE** | **SOURCE** | **IDENTIFIER** | **RRID** |
| --- | --- | --- | --- |
|  |  |  |  |
| **Antibodies** |  |  |  |
| β-actin | Cell Signaling Technology | 5125 | AB_1903890 |
| β-tubulin | Cell Signaling Technology | 2146 | AB_2210545 |
| Axl (C89E7) | Cell Signaling Technology | 8661 | AB_11217435 |
| Phospho-Axl (Tyr702) (D12B2) | Cell Signaling Technology | 5724 | AB_10544794 |
| CAD | Cell Signaling Technology | 93925 | AB_2750933 |
| Phospho-CAD Serine 1859 | Cell Signaling Technology | 12662 | AB_2750934 |
| GAS6 (D3A3G) | Cell Signaling Technology | 67202 | AB_2799720 |
| H2A.X | Cell Signaling Technology | 7631 | AB_10860771 |
| Phospho-H2A.X Serine 139 | Cell Signaling Technology | 9718 | AB_2118009 |
| PTEN (D4.3) XP | Cell Signaling Technology | 9188 | AB_2253290 |
| S6 | Cell Signaling Technology | 2217 | AB_331355 |
| Phospho-S6 Ribosomal Protein (Ser235/236) IHC | Cell Signaling Technology | 4858 | AB_916156 |
| Phospho-S6 Ribosomal Protein (Ser240/244) (D68F8) | Cell Signaling Technology | 5364 | AB_10694233 |
| S6K1 | Cell Signaling Technology | 2708 | AB_390722 |
| S6K2 | R & D Systems | AF2987 | AB_10890762 |
|  |  |  |  |

| **Chemicals, Lipids, and Recombinant proteins** |  |  |  |
| --- | --- | --- | --- |
| ^13^C-glucose | Cambridge Isotope Laboratories | CLM-8367-PK |  |
| B-27™ Supplement | Gibco | 12587-010 |  |
| Blasticidine S hydrochloride | Sigma-Aldrich | 15205 |  |
| BMS-777607 | Selleckchem | S1561 |  |
| Calcein green | Corning | 354217 |  |
| Cytotox red | Sartorius | 4632 |  |
| DMEM | Corning | 10-017-CV |  |
| DMSO | Sigma-Aldrich | D2650 |  |
| Fetal Bovine Serum | Gibco | 16000044 |  |
| Formalin | Fisher | SF99-4 |  |
| Human EGF | IrvineScientific | 100-26 |  |
| Human FGF-basic 154 | IrvineScientific | 100-146 |  |
| Human Gas6 Recombinant Protein | R&D Systems | 885-GSB |  |
| L-glutamine | Cytiva | SH30034.01 |  |
| L-α-phosphatidylserine | Avanti Polar Lipids | 840032 |  |
| Lipofectamine 3000 | Invitrogen | L3000 |  |
| Luciferin | Gold Biotechnology | LUCK |  |
| LY-2584702 | Selleckchem | S7698 |  |
| LY-2584702 tosylate | Selleckchem | S7704 |  |
| Matrigel | Corning | 354230 |  |
| MycoAlert | Lonza | LT07 |  |
| Neurobasal™ Medium | Gibco | 21103049 |  |
| NeuroCult NS-A Proliferation Kit | StemCell | 5751 |  |
| OptiMEM | Gibco | 31985070 |  |
| PEG300 (NF) | Spectrum | PO108 |  |
| Penicillin/ Streptomycin | Cytiva | SV30010 |  |
| PF-4708671 | Selleckchem | S2163 |  |
| Phophatase inhibitor, PhosSTOP | Sigma-Aldrich | 4906845001 |  |
| Protease inhibitor, cOmplete | Sigma-Aldrich | 11836153001 |  |
| Puromycin dihydrochloride | Sigma-Aldrich | P9620 |  |
| PVDF membrane | Amersham | GE10600023 |  |
| Stemsol (USP DMSO) | Protide | PP1250 |  |
| TRIzol | Invitrogen | 15596026 |  |
|  |  |  |  |
| **Experimental models: Cell lines** |  |  |  |
| LN229 | ATCC | CRL-261 |  |
| U87MG | ATCC | HTB-14 |  |
| 293T cells | ATCC | CRL-3216 |  |
| JHH136 | Soma Sengupta, University of North Carolina, gift of  Gregory Riggins, Johns Hopkins University |  |  |
| Mayo59 | Jann Sarkaria, Mayo Clinic, Rochester, MN | GBM59 |  |
| U87MG-GFP-Luc | Atsuo Sasaki Lab, University of Cincinnati | N/A |  |
| U87MG Dox-inducible PTEN | Atsuo Sasaki Lab, University of Cincinnati | N/A |  |
|  |  |  |  |
| **CRISPR Knock out cell lines** |  |  |  |
| gRNA Non-targeting control | Made in lab | N/A |  |
| sgS6K1 ex2 LN229 | Made in lab | N/A |  |
| sgS6K1 ex5 LN229 | Made in lab | N/A |  |
| sgS6K2 ex5 LN229 | Made in lab | N/A |  |
| sgS6K2 ex9 LN229 | Made in lab | N/A |  |
| sgS6K1 ex2 U87MG-GFP-Luc | Made in lab | N/A |  |
| sgS6K1 ex5 U87MG-GFP-Luc | Made in lab | N/A |  |
| sgS6K2 ex5 U87MG-GFP-Luc | Made in lab | N/A |  |
| sgS6K2 ex9 U87MG-GFP-Luc | Made in lab | N/A |  |
|  |  |  |  |
| **Plasmids** |  |  |  |
| RPS6KB1 gRNA | Addgene | 75613 | Addgene_75613 |
| RPS6KB1 gRNA | Addgene | 75614 | Addgene_75614 |
| RPS6KB2 gRNA | Addgene | 76057 | Addgene_76057 |
| RPS6KB2 gRNA | Addgene | 76058 | Addgene_76058 |
| lenti Cas9 Blasticidin | Addgene | 52962 | Addgene_52962 |
| pLX304 Luciferase-V5 blast | Addgene | 98580 | Addgene_98580 |
| Non-targeting control gRNA (BRDN0001149198) | Addgene | 80248 | Addgene_80248 |
|  |  |  |  |
| **siRNA** |  |  |  |
| Human RPS6KB1(6198) smart pool, 5 nmol | Horizon Discovery | M-003616-03-0005 |  |
| Human RPS6KB2 (6199) smart pool, 5 nmol | Horizon Discovery | M-004671-01-0005 |  |
| Human PTEN smart pool, 5 nmol (5728) | Horizon Discovery | M-003023-02-0010 |  |
| siGENOME Non-Targeting Control siRNA #1 | Horizon Discovery | D-001210-01 |  |
| siGENOME Human RPS6KB2 siRNA #2 | Horizon Discovery | D-004671-02 |  |
| siGENOME Human RPS6KB2 siRNA #3 | Horizon Discovery | D-004671-03 |  |
|  |  |  |  |
| **Animals for Preclinical Studies** |  |  |  |
| C57B/6J | Jackson Laboratories | 000664 |  |
| NOD SCID gamma | Jackson Laboratories | 005557 |  |
| Athymic Nude | Charles River | 553 |  |
| NOD CRISPR Prkdc IL2 gamma | Charles River | 572 |  |
